# Sense of agency, associative learning, and schizotypy

**DOI:** 10.1016/j.concog.2011.01.002

**Published:** 2011-09

**Authors:** James W. Moore, Anthony Dickinson, Paul C. Fletcher

**Affiliations:** a Department of Psychiatry, Brain Mapping Unit, University of Cambridge, Downing Site, Cambridge, United Kingdom; b Department of Experimental Psychology, University of Cambridge, Cambridge, United Kingdom

**Keywords:** Voluntary action, Sense of agency, Prediction error, Associative learning, Schizotypy, Schizophrenia

## Abstract

Despite the fact that the role of learning is recognised in empirical and theoretical work on sense of agency (SoA), the nature of this learning has, rather surprisingly, received little attention. In the present study we consider the contribution of associative mechanisms to SoA. SoA can be measured quantitatively as a temporal linkage between voluntary actions and their external effects. Using an outcome blocking procedure, it was shown that training action–outcome associations under conditions of increased surprise augmented this temporal linkage. Moreover, these effects of surprise were correlated with schizotypy scores, suggesting that individual differences in higher level experiences are related to associative learning and to its impact on SoA. These results are discussed in terms of models of SoA, and our understanding of disrupted SoA in certain disorders.

## 1 Introduction

The feeling of producing events through one’s own intentional behaviour is referred to as ‘Sense of Agency’ (SoA), and has been the focus of much research. A key challenge is to elucidate the nature of processes underpinning this experience.

Various findings point towards the importance of learning in the context of SoA. For example, SoA appears to be sensitive to the causal relation between actions and outcomes (Cravo, Claessens, & Baldo, 2009; Franck et al., 2001; Moore & Haggard, 2008; Moore, Lagnado, Deal, & Haggard, 2009). Furthermore, pharmacological manipulations suggest that SoA is dependent on neural circuits implicated in instrumental learning (Moore et al., 2010). Certain theoretical models of SoA also point towards the importance of learning. For example, it has been suggested that SoA is informed by sensorimotor predictions generated as part of the normal system of motor control (Blakemore, Wolpert, & Frith, 2002; Frith, Blakemore, & Wolpert, 2000). Given that valid predictions are the hallmark of successful learning, this model, at least implicitly, recognises the contribution of learning. In light of the apparent importance of learning for SoA it is surprising that the precise mechanisms of that learning have received little attention. This is something the current study wished to address.

Associative mechanisms may be one way in which learning about action–outcome relations is achieved. Central to traditional theories of associative learning is the role of surprise or prediction error (Mackintosh, 1975; Pearce & Hall, 1980; Rescorla & Wagner, 1972). According to these theories, more learning occurs when the outcome is unexpected. The importance of surprise for learning can be demonstrated by blocking – traditionally regarded as the canonical test of the involvement of associative mechanisms (Dickinson, 1981). In a standard blocking paradigm (see Kamin, 1969), pre-training a cue(X)-outcome association blocks the subsequent formation of a novel cue(Y)-outcome association when that cue(Y) is subsequently trained in compound with the original cue(X). The blocking of cue(Y)-outcome associations is thought to occur because the outcome is already fully predicted by cue(X) during compound training: there is no surprise and therefore little new learning.

The importance of associative mechanisms for *instrumental* learning has also been demonstrated. Flach, Osman, Dickinson, and Heyes (2006) developed an outcome blocking procedure in which the *outcomes* competed for association with the action. This differs from traditional blocking designs, described above, in which preceding events compete for association with a subsequent event. In their design, a pre-training stage established action–outcome associations between two actions (right and left key presses) and two visual outcomes (coloured patches). In a compound training stage, these actions were then paired with a compound of two outcomes (a coloured patch and a tone). For one action, the visual outcome was the same as that learned during pre-training so that only the auditory outcome was unexpected. For the other action both outcomes were entirely novel and therefore *surprising*. They found that the strength of action–outcome associations was stronger when both outcomes during training were surprising. In the present study we use Flach et al’s outcome blocking procedure to examine the influence of surprise on the learning of action–outcome relations, and the impact this has on SoA.

**Fig. 1:**
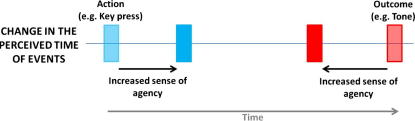
Sense of agency is associated with changes in the perceived time of actions and outcomes. Increased sense of agency is associated with later awareness of action and earlier awareness of outcome.

Our measure of SoA was an implicit one based on the perceived temporal relation between actions and outcomes. Previous research by Haggard, Clark, and Kalogeras (2002) found that situations that elicit SoA are associated with a subjective compression of the interval between an action and its sensory outcome. In an agency condition, in which their actions produced tones, participants judged the time of an action or the time of the subsequent tone, in separate blocks of trials. Actions were perceived as occurring later in time, i.e. shifted towards the tone, when compared to a non-agency condition in which participants’ actions did not produce tones. In addition, a tone that followed the action was perceived as occurring earlier, i.e. shifted in time towards the action that caused it (again, in comparison to a non-agency condition involving tones but no actions). Critically, these shifts were found for voluntary actions but not when the tone was caused by an involuntary movement. Therefore, increased SoA is associated with a later awareness of the action (e.g. a key press), and an earlier awareness of the outcome (e.g. a tone; see Fig. 1): actions and outcomes are bound together in time. This effect has been consistently replicated (see Engbert & Wohlschläger, 2007; Engbert, Wohlschläger, & Haggard, 2008; Moore, Wegner, & Haggard, 2009; Tsakiris & Haggard, 2003), and has also been shown to correlate with explicit higher-order changes in the SoA, as measured using subjective rating scales (Ebert & Wegner, 2010; Moore & Haggard, 2010). This temporal binding therefore offers a precise, implicit measure of SoA.

In the present experiment, participants performed the same outcome blocking procedure developed by Flach et al. (2006). The only change was to the test phase, where we replaced Flach et al.’s measure of instrumental learning with our temporal binding measure of SoA. We predicted that binding at test would be augmented for actions that were previously trained with entirely novel, and therefore *surprising*, outcomes.

In addition, we sought to relate the effects of surprise on binding to individual difference measures. In particular we focussed on schizotypy scales designed to quantify perceptions and beliefs that may be akin to those found in schizophrenia. Schizophrenia may be characterised by a disturbance in, or confusion over, one’s SoA, something that has also been associated with higher scores on schizotypy scales (e.g. Asai, Sugimori, & Tanno, 2008). This is especially relevant to the current experimental manipulation given that recent models of the symptoms of schizophrenia emphasise the role of disturbed prediction error generated by surprising events (Corlett, Honey, & Fletcher, 2007; Corlett et al., 2007; Fletcher & Frith, 2009; Gray, Feldon, Rawlins, Hemsley, & Smith, 1991; Kapur, 2003; Murray et al., 2008). Although the link between schizotypy and schizophrenia is not fully established, given the *putative* link, the schizotypy dimension is therefore relevant when considering individual differences on effects of surprise on SoA.

## 2 Methods

### 2.1 Participants

Sixteen right-handed participants were recruited to the study (11 female; mean age = 25 years). One participant was excluded based on failure to follow task instructions. All participants provided informed consent. Research was approved by University of Cambridge Psychology Research Ethics Committee.

### 2.2 Outcome blocking procedure

**Fig. 2:**
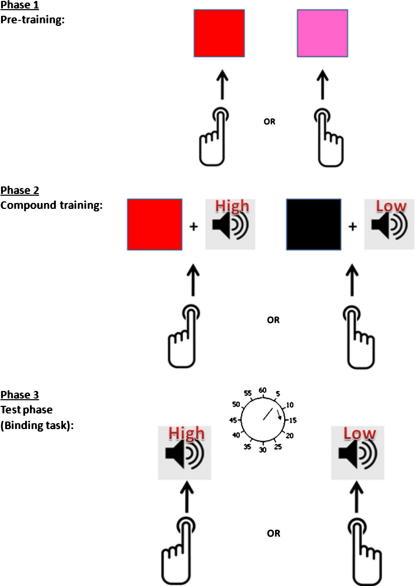
The training and test phases in Experiment 2 (following Flach et al. (2006)). See text for details.

The experiment consisted of three sequential phases (see Fig. 2 for schematic).

**Fig. 3:**
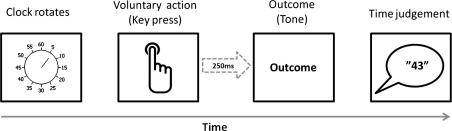
The trial structure used in the test phase (following Haggard et al. (2002)). Clock begins rotating at start of each trial. Participant presses one of two keys whenever they feel the urge to do so. After a brief delay (250 ms) the tone is presented. After a random period of time the clock hand then stops rotating. The participant is then prompted to make their time judgement i.e. the time (1–60) that corresponds to the position of the hand on the clock face when they pressed the key or, in a separate block of trials, when they heard the tone.

Phase 1 – *Pre-training*: Participants freely pressed one of two keys on each trial at a time of their own choosing. The ‘left key’ was pressed with the left index finger and the ‘right key’ was pressed with right index finger. These key presses caused a flash of colour to be displayed in a rectangular box on the centre a computer screen in front of them (duration: 200 ms) after a delay of 250 ms. The colour was either red or pink. The mapping of colour to key press was constant throughout this phase, and was fully counterbalanced across participants. This phase consisted of 80 trials.
Phase 2 – *Compound training*: Participants freely pressed one of two keys on each trial at a time of their own choosing. Key presses caused both a flash of colour and a tone to be presented simultaneously (duration: 200 ms) after a delay of 250 ms. The colour was either one that had been presented during *pre-training* (red or pink), or a new colour (black). The tone was either a high or low pitch tone. The mapping of the compound stimulus to key press was constant throughout this phase, and assignment of the auditory stimuli to the two actions was fully counterbalanced across participants. The presentation of stimuli ensured that for each participant one key press always caused an entirely novel stimulus compound (surprise condition), whereas the other key press always caused a stimulus compound in which the colour had been previously associated with the same key press in the pre-training phase (expected condition). This phase consisted of 80 trials.
Phase 3 – *Test*: This test was based on previous studies on the temporal experience of action–outcome associations (e.g. Haggard et al., 2002) and is shown schematically in Fig. 3. Participants freely pressed one of the two keys when they felt the urge to do so. Pressing the key caused, after a delay of 250 ms, the same tone that had followed that key press in the *compound training phase*. Time judgements were made using a clock hand rotating around a clock face. The clock face was located centrally on the computer screen and was marked at conventional 5 min intervals. The clock hand rotated rapidly around the clock face at a speed of one revolution every 2.56 s. The clock hand continued to rotate for a random period of time after the presentation of the tone. When the clock hand stopped rotating, participants were prompted to verbally report the position of the clock hand on the clock face either when they had pressed the key, or when they had heard the subsequent tone. Participants performed one block of action judgements and one block of tone judgements. Each block consisted of 40 trials, and their order was counterbalanced. For each participant, the mapping of tone to key press was kept constant, but was counterbalanced across participants. *Surprise* action–tone pairings were those in which the tones had previously been presented in the novel stimulus compound, *expected* action–tone pairings were those in which the tones had previously been presented with a pre-trained colour.

Mean judgement errors for each trial type (*surprise*/*expected*) and for each judgement type (*Action*/*Tone*) were calculated for each participant. Mean judgement errors are the mean of the trial-by-trial differences between judged time and actual time of event onset (judged time minus actual time). Later action awareness and earlier tone awareness reflect enhanced SoA, which is manifest by a positive shift in the mean judgment error for the response and a negative shift for the tone. For the analysis, test trials were divided into *early* (first 20 trials) and *late* (final 20 trials), as previous research has shown that effects of training may change over time (Flach et al., 2006; Moran, Al-Uzri, Watson, & Reveley, 2003).

### 2.3 Schizotypy measures

The *Perceptual Aberration Scale* (Chapman, Chapman, & Raulin, 1978) is a 35-item scale assessing distortions in the perceptual experience of one’s own body and surroundings (e.g. “Occasionally I have felt as though my body did not exist”).

The *Magical Ideation Scale* (Eckblad & Chapman, 1983) is a 30-item scale assessing beliefs that are inconsistent with the cultural standard (e.g. “I have felt that I might cause something to happen just by thinking too much about it”).

The *Peters Delusion Inventory* (Peters, Joseph, Day, & Garety, 2004) is a 21-item scale designed to measure delusional ideation in the normal population (e.g. “Do your thoughts ever feel alien to you in some way?”). When an item is endorsed, three five-point scales exploring distress, preoccupation, and beliefs are then completed.

## 3 Results

### 3.1 Effect of surprise on binding

We analysed subjective time estimates of actions (key presses) and outcomes (tones) on early and late test trials as a function of the surprisingness of that association during training. Test trials were divided into *early* (first 20 trials) and *late* (final 20 trials), as previous research has shown that effects of training may change over time (Flach et al., 2006; Moran et al., 2003).

**Fig. 4:**
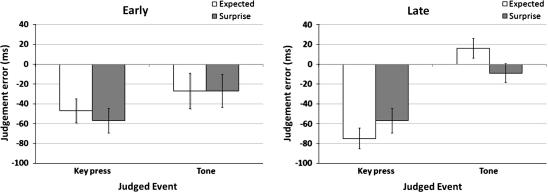
Mean judgment errors in ms (SD across subjects) for ‘expected’ and ‘surprise’ training (early vs. late). Zero on the *y*-axis represents actual event onset. Later action (key press) awareness and earlier outcome (tone) awareness reflects increased binding. This is indicative of increased SoA (see text).

Mean judgement error of action and tone time estimates for trials (early vs. late) are shown in Fig. 4. Inspection of the graph suggests that training under conditions of augmented outcome surprise enhanced the temporal binding of actions and tones at test (later action judgements and earlier tone judgements), but only on late test trials.

To test this, a repeated measures ANOVA was conducted on factors of *time* (early, late), *training* (surprise, expected), and *judged event* (action, tone). There were no significant main effects of *time*, *training*, or *judged event*, and no significant two-way interactions between these factors. However, there was a significant three-way interaction between all three factors, *F*(1, 14) = 7.26, *p* < .05 (partial eta-squared = .34).

Follow-up repeated measures ANOVA analyses (*training* × *judged event*) were performed for both *early* and *late* test trials. There were no significant main effects and no interaction for *early* test trials. For *late* test trials there was a significant main effect of *judged event*, *F*(1, 14) = 7.53, *p* < .05 (partial eta-squared = .35). Tones were perceived significantly later than actions (relative to actual event onset).

More importantly, there was a significant interaction between *training* and *judged event* for *late* test trials, *F*(1, 14) = 11.20, *p* < .01 (partial eta-squared = .44). Follow-up paired-samples t-tests showed that training under a condition of augmented surprise was associated with significantly later action awareness compared with expected trials, *t*(14) = 2.25, *p* < .05 (2-tailed; Cohen’s *d* = .24). There was no significant effect of training on the awareness of tones, *t*(14) = 1.75, *p* = .10 (2-tailed; Cohen’s *d* = .15). This suggests that the effect of training with surprising outcomes on SoA is limited to *late* test trials and is most pronounced for actions.

### 3.2 Schizotypy and the effects of surprise on binding

The mean schizotypy scores were as follows: PDI = 77 (range: 14–131; Cronbach’s Alpha: .76); Chapman Magical Ideation = 8 (range: 3–15; Cronbach’s Alpha: .80); Chapman Perceptual Aberration = 7 (range: 0–21; Cronbach’s Alpha: .88). For purposes of comparison, Table 1 shows example mean scores (and ranges where available) from healthy controls and patients with schizophrenia tested in previous larger-scale studies.

We then assessed the relationship between schizotypy and the effects of surprise on binding. The magnitude of the outcome surprise effect was calculated for early and late test trials. To assess this magnitude, we first generated a composite measure of binding (tone judgement error minus action judgement error) for ‘expected’ and ‘surprise’ training on early and late test trials. The magnitude of the outcome surprise effect represented the difference in the surprise and expected composite measures (surprise composite measure minus expected composite measure). The more positive this difference, the greater the surprise effect.

**Fig. 5:**
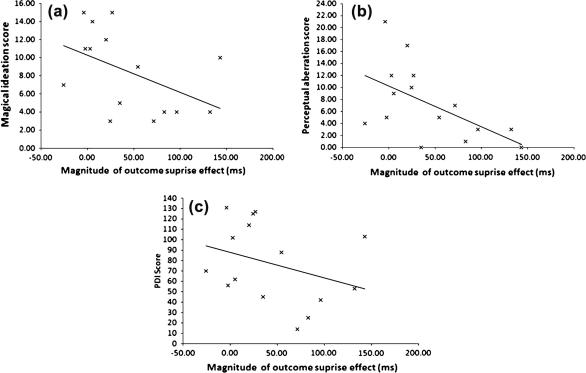
Scatter plots depicting relation between magnitude of outcome surprise effect and (a) Magical Ideation scores, (b) Perceptual Aberration scores, and (c) PDI scores.

Schizotypy scores on each of the scales were correlated with magnitude of the surprise effect (see Fig. 5), using the non-parametric Spearman’s rank order correlation (1-tailed). This analysis was limited to late test trials as the surprise effect was only observed on these trials. Significant negative correlations were found for the relationship between the magnitude of the surprise effect and Magical Ideation (rho = −.52, *p* < .05), and Perceptual Aberration (rho = −.62, *p* < .01). There was also a near significant negative correlation between the magnitude of the surprise effect and the Peters Delusion Inventory (rho = −.43, *p* = .057). These correlations suggest that the effects of surprise during training are reduced in those scoring higher on the schizotypy scales.

## 4 Discussion

This experiment found that surprise exerts an influence over SoA. Using an outcome blocking procedure we were able to show that training with more surprising outcomes augmented binding at test (at least on later test trials), indicative on an increased SoA This result confirms the importance of learning in the context of SoA. Moreover, it suggests that associative mechanisms may be involved. Finally, the effects of surprise on SoA were correlated with schizotypy scores, suggesting that individual differences in associative learning may be related to higher level experiences of the world that resemble (to varying degrees) those associated with schizophrenia.

### 4.1 Associative learning and SoA

Despite the fact that the role of learning is recognised in empirical (e.g. Moore et al., 2009) and theoretical (e.g. Frith, 1992; Frith et al., 2000) work on SoA, the nature of this learning has, rather surprisingly, received little attention. Blocking is traditionally regarded as a canonical demonstration of associative learning mechanisms (Dickinson, 1981). Therefore, the sensitivity of binding to our outcome blocking procedure strongly implies the involvement of associative mechanisms in producing SoA.

Outcome prediction is thought to play a key role in generating SoA (Blakemore et al., 2002; Frith et al., 2000). Indeed, a previous study using the binding task found that the magnitude of binding was sensitive to the strength of the outcome prediction (Moore & Haggard, 2008). The present experiment complements this finding by shedding light on the learning mechanism that enables such predictions. Specifically, our data suggest that the learning necessary for outcome prediction is achieved, at least in part, by associative mechanisms.

The role of surprise has been emphasised in previous theoretical accounts of agency experience. For example, Frith and colleagues have suggested that SoA is decreased when the outcome is surprising (e.g. Frith et al., 2000; see also, Barnier, Dienes, and Mitchell (2008) for a discussion of the role of surprise in the experience of control under hypnosis). Whilst the present results support the role of surprise in SoA, they offer a rather different view of its contribution to SoA, namely that in the long-run surprise may augment SoA. Although we shall not recapitulate here the extensive discussion of associative explanations concerning the role of surprise in outcome blocking offered by Flach et al. (2006), the present results are compatible with the attentional account of Pearce and Hall (1980). They suggest that the attention paid to an antecedent event, in this case a response, is determined by whether or not that event has previously been associated with a prediction error generated by surprising outcomes. Therefore, the attention paid to the responses should have decreased during pre-training as their respective visual outcomes came to be anticipated. The introduction of the auditory outcome during compound training would have been surprising, resulting in partial restoration of attention to the response and some response-tone learning. However, the additional presence of the unexpected visual outcome in the surprise condition would have produced an even greater prediction error and restoration of attention to the response, thereby supporting even more sustained action–outcome learning.

We propose that so long as an agent recognises itself as the originator of an event by attending to its own behaviour, the strength of action–outcome associations will be updated. A corollary of this view is that individual differences in the extent to which an agent recognises itself as the originator of an event will lead to different error-driven learning effects. If someone has a tendency to attribute error to an external source they will be less likely to use that error to update their own causal beliefs. This may explain attenuated blocking effects on our task in participants scoring higher on the schizotypy scales: in the condition of augmented surprise they fail to increase the strength of the action–outcome association, possibly because they fail to register the fact that they are responsible for the unexpected event (maybe in these participants error itself promotes a stronger feeling that they were not the originator of the unexpected event).

The putative involvement of associative mechanisms is consistent with a previous result showing that SoA (as measured by binding) was altered by manipulations of dopamine availability in patients with Parkinson’s Disease (PD; Moore et al., 2010). Using an ON–OFF drug withdrawal design, it was found that although binding in PD patients OFF their dopaminergic medication was similar to controls, binding in the same PD patients ON medication was significantly *increased*. By coding prediction error, dopaminergic systems have a special role in associative learning. It was therefore suggested that the exaggerated binding found in PD patients ON medication could have been driven by a change in dopaminergic prediction error signalling, augmenting the strength of action–outcome associations. This suggestion is bolstered by the observation in the current paper that SoA is indeed likely to have an associative basis. Furthermore, given the hypothesised role of disrupted prediction error signalling in the genesis of schizophrenia, our observation that SoA has an associative basis, and which in turn may be linked to dopamine function, is also relevant to understanding impaired SoA associated with that disorder (see Section 4.2).

The contribution of associative learning mechanisms to SoA has further implications for theoretical accounts of SoA. At the very least this finding emphasises the need to (a) recognise the dynamic nature of SoA and (b) consider the specific mechanisms enabling learning in the context of SoA. Perhaps more fundamentally, the putative role of associative learning mechanisms strongly encourages us to reconsider the contribution of automatic low level processes to this higher level experience. Furthermore, the aforementioned link between mechanism (associative learning) and neurobiology (dopamine) in the context of SoA is appealing, offering the possibility of a more comprehensive account of SoA and its disturbance in certain disorders.

### 4.2 Surprise, SoA, and schizotypy

We found significant correlations between the effect of surprise on SoA and scores on schizotypy scales. Specifically, the effect of surprise during training was reduced in highly schizotypal individuals. Given the similarity between the procedure used in this experiment and traditional blocking procedures, this result is consistent with previous investigations of blocking and schizotypy (e.g. Moran et al., 2003).

Although the relationship between schizotypy and schizophrenia is yet to be fully explicated, it is thought that schizotypal characteristics are likely to represent cognitive vulnerability for the disorder (Chapman, Chapman, & Kwapil, 1994; Chapman, Chapman, Kwapil, Eckblad, & Zinser, 1994; Langdon & Coltheart, 1999). In this respect our data may be in keeping with previous work emphasising the link between disrupted associative learning and schizophrenia. According to this perspective, disrupted prediction error signalling is thought to underpin many of the signs and symptoms of schizophrenia. Moreover, such disruption is thought to be of central importance to the genesis of psychotic illness (Corlett, Murray et al., 2007), and as such, studies linking schizotypy to disrupted prediction error signalling may be especially informative (see, for example, Corlett et al., 2009).

These data show that participants scoring higher on schizotypy scales were less sensitive to prior associative history. As discussed above, this may be linked to differences in the way that these individuals process surprising events during training. Such reduced sensitivity may underlie the different experiences of, and beliefs about, the world that are reflected in high scores on the schizotypy scales. However, owing to the intimate relation between perception and belief, and the fact that both may be underpinned by a common mechanism involving the minimisation of prediction error (Fletcher & Frith, 2009), we would not expect differences in the strength of the association between sensitivity to prior associative history and the perception (Chapman Perceptual Aberration) or belief (Peters Delusion Inventory and Chapman Magical Ideation) schizotypy scales. This was indeed what we found.

Although such schizotypy-related differences on associative learning tasks have been demonstrated before, to our knowledge this is the first study demonstrating such deficits on an instrumental task. Compared with Pavlovian paradigms (where performance is based on learning relations between *external* stimuli), instrumental paradigms may be more relevant to symptoms of schizophrenia in which willed action and the SoA may be altered (as is the case in delusions of control). Our findings raise the possibility that the differential levels of sensitivity to surprise leads to a profoundly different experience of our actions on the world. The choosing and carrying out of these actions is absolutely critical to how we sample, and therefore learn about, the world (Friston, 2009; Friston, Daunizeau, Kilner, & Kiebel, 2010). A corollary of this would be that such a fundamental variation might lead to a very different experience of the world and, correspondingly, altered inferences about its causal structures and one’s own capacity to alter it.

### 4.3 Limitations

The fact that training effects were only found on late test trials was unexpected. In the initial test trials of the expected condition there would have been a (surprising) omission of an expected visual effect. If surprise influences the degree of binding (as implied by our overall results) it may have initially augmented the magnitude of binding on early test trials, therefore masking the effects of prior associative learning history. With further testing, the omission of the visual outcome would have no longer been surprising thereby attenuating this ‘local’ surprise effect in the expected condition and allowing the sustained associative surprise effect on the SoA to be observed in the surprise condition.

Given the relatively small sample size one should be careful in drawing overly strong conclusions regarding the correlation between schizotypy and the effects of surprise on our task. However, it is encouraging that significant (or near significant) negative correlations were observed on all three schizotypy scales, which we believe is highly suggestive of a genuine association.

As stated above, the relationship between schizotypy and schizophrenia is yet to be fully explicated. In this way, one should be cautious when discussing schizotypy findings in terms of their relevance for schizophrenia. However, it has been repeatedly shown on experimental tasks that the behaviour of individuals scoring high on schizotypy scales resembles that of patients with schizophrenia (Peters, 2010), and our data provide further demonstration of this concordance. We would like to further suggest that, given sufficient confidence in the relationship between schizotypy and schizophrenia, the schizotypy dimension offers an invaluable research tool, allowing us to explore patterns of perception, experience and cognition redolent of schizophrenia, in a sample that is free of medication and hospitalisation (both of which can be deeply problematic confounds in patient samples).

Finally, certain limitations of our test phase should be acknowledged. Firstly, the test phase was fairly short, consisting of 40 trials per judgement type (action or tone). However, the fact that learning effects could be detected would suggest that the duration of the test phase sufficient. Secondly, the test phase was divided into early and late trials. With such a division one inevitably ends up treating two trials that are positioned close together in time, as being early or late, based on whether they were the first or last 20 trials of the test block. Although this may be a rather imprecise way of characterising learning, it nevertheless still allows us to determine the presence of any general changes in behaviour during the course of the test phase. A priori, we felt that it was important to assess these possible changes owing to previous studies suggesting that the effects of learning may change during the test phase.

### 4.4 Concluding remarks

Our study has shown that learning underpinning SoA (as measured by binding) may be governed, at least in part, by associative mechanisms. This novel finding has implications for theoretical accounts of SoA and for understanding disrupted SoA in certain disorders. This is confirmed by the fact that the effect of surprise on binding was correlated with scores on schizotypy rating scales. Owing to the possible relationship between schizotypy and schizophrenia, this suggests that models emphasising the role of aberrant (dopaminergic) prediction error signalling in the emergence of psychosis may have validity in understanding the nature of experience in schizotypy.

## Figures and Tables

**Table 1:** Example mean scores (with range where available) on the schizotypy scales that were used in the present study. Data are from controls and patients with schizophrenia tested in previous studies.

	Peters Delusion Inventory (from Peters et al. (2004))	Magical Ideation Scale (from Peters, Joseph, and Garety (1999))	Perceptual Aberration Scale (from Chapman et al. (1978))
Healthy controls	58.9 (0–291)	11.5 (5–25)	5.14 (N/A)
Schizophrenia patients	130.5 (0–317)	13.7 (5–25)	7.68 (N/A)
